# Selenium uptake, translocation, subcellular distribution and speciation in winter wheat in response to phosphorus application combined with three types of selenium fertilizer

**DOI:** 10.1186/s12870-023-04227-6

**Published:** 2023-04-27

**Authors:** Caixia Hu, Zhaojun Nie, Huazhong Shi, Hongyu Peng, Guangxin Li, Haiyang Liu, Chang Li, Hongen Liu

**Affiliations:** 1 Resources and Environment College, Henan Agricultural University, Jinshui District, No. 63, Nongye RoadHenan Province, Zhengzhou, 450002 China; 2 Department of Chemistry and Biochemistry, Texas Tech University, Lubbock, TX 79409 USA

**Keywords:** Winter wheat, Phosphorus, Selenium, Uptake, Translocation, Subcellular distribution, Se species

## Abstract

**Background:**

Selenium (Se) deficiency causes a series of health disorders in humans, and Se concentrations in the edible parts of crops can be improved by altering exogenous Se species. However, the uptake, transport, subcellular distribution and metabolism of selenite, selenate and SeMet (selenomethionine) under the influence of phosphorus (P) has not been well characterized.

**Results:**

The results showed that increasing the P application rate enhanced photosynthesis and then increased the dry matter weight of shoots with selenite and SeMet treatment, and an appropriate amount of P combined with selenite treatment increased the dry matter weight of roots by enhancing root growth. With selenite treatment, increasing the P application rate significantly decreased the concentration and accumulation of Se in roots and shoots. P_1_ decreased the Se migration coefficient, which could be attributed to the inhibited distribution of Se in the root cell wall, but increased distribution of Se in the root soluble fraction, as well as the promoted proportion of SeMet and MeSeCys (Se-methyl-selenocysteine) in roots. With selenate treatment, P_0.1_ and P_1_ significantly increased the Se concentration and distribution in shoots and the Se migration coefficient, which could be attributed to the enhanced proportion of Se (IV) in roots but decreased proportion of SeMet in roots. With SeMet treatment, increasing the P application rate significantly decreased the Se concentration in shoots and roots but increased the proportion of SeCys_2_ (selenocystine) in roots.

**Conclusion:**

Compared with selenate or SeMet treatment, treatment with an appropriate amount of P combined with selenite could promote plant growth, reduce Se uptake, alter Se subcellular distribution and speciation, and affect Se bioavailability in wheat.

**Supplementary Information:**

The online version contains supplementary material available at 10.1186/s12870-023-04227-6.

## Background

Selenium (Se) is an essential microelement for humans and animals and plays important roles under oxidative stress-related conditions and in immune system support and disease prevention [1, 2]. In recent decades, hidden hunger caused by Se deficiency has been commonly found worldwide [3], resulting in health disorders such as muscle syndrome, Keshan disease, liver disease, cognitive impairment and many cancers [4, 5]. Excessive Se intake is also harmful, resulting in health disorders such as hair loss, nervous system disorders and paralysis [6]. The World Health Organization suggests a daily intake of Se of 50–55 μg for adults. However, it has been estimated that the Se intake amount of approximately 0.7 billion people in the world is lower than the recommended value [7]. Studies have also shown that 72% of soil is Se deficient in China [8]. Therefore, Se-enriched fertilizers were applied to improve Se contents in edible plant parts and increase Se intake for humans living in Se-deficient areas [9–11].

It is well known that Se availability is influenced by pH, Eh, and Se species in soil [12, 13]. Se exists in soil in four oxidation states (-II, 0, IV and VI) [14], of which selenide (-II) and Se (0) are difficult for plant roots to absorb. Selenite (IV) is the main form of Se in acidic and neutral soils (pE + pH < 7.5), but selenate (VI) mainly exists in oxidized and alkaline soils (pE + pH > 15). Plants can uptake both selenite and selenate, most of which is converted into selenocystine (SeCys_2_) and selenomethionine (SeMet) [15]. Compared to selenite and selenate, organic Se is safe and beneficial for plants [16–18]. SeMet can be absorbed by plants through amino acid transporters [17]. The translocation and metabolism of selenate, selenite and organic Se showed differences after absorption in plants [19, 20]. Furthermore, different forms of Se influence Se concentration, species and distribution [21]. For example, selenite mainly accumulates in roots, but selenate accumulates in shoots [11, 22, 23]. SeMet is the dominant organic Se in plants, and it is translocated from roots to shoots via peptide transporter (*NRT1.1B*) in plants [24, 25]. Organic Se can be absorbed into the phloem and then translocated to grains via the stem, while most inorganic Se is translocated through the xylem to grains [26].

It has been reported that selenite is absorbed by roots via passive diffusion or phosphate transporters [11, 23]. Studies have demonstrated that the translocation of selenite between different plant organs is promoted by phosphorus (P) application [27]. P fertilizer increased the Se concentration in the soil and then promoted Se absorption and accumulation in rice [28]. However, several studies have shown that there is an antagonistic effect between phosphate and selenite. Therefore, the interaction of phosphate and selenite still needs to be verified. Generally, phosphate has little effect on the uptake of selenate due to their chemical dissimilarities. P starvation had no effect on selenate uptake but decreased selenate concentration in the xylem sap of selenate-treated plants in hydroponic experiments [11]. Some studies reported that P application increased the utilization of selenate and then increased the Se contents in plants [29, 30]. Compared with the numerous studies on inorganic Se, few studies have focused on the uptake of organic Se influenced by P application in plants.

Generally, inorganic Se (selenite and selenate) and selenoamino acids (SeMet, SeCys_2_ and Se-methyl-selenocysteine (MeSeCys)) are present in plants [13, 22, 31]. Compared with inorganic Se, organic Se is safer to human health. Studies have noted that different Se sources, P nutrient states and crop varieties have a significant influence on Se species in plants [13, 20, 32, 33]. Selenite can be quickly assimilated in roots and then converted into organic Se (SeMet, SeCys or Selenomethionine selenoxide (SeOMet)); however, selenate can be detected in shoots due to its quick mobility during xylem transport [11]. Studies have demonstrated that P deficiency increased the proportion of MeSeCys in rice, but MeSeCys was not detected when P was added to the nutrient solution [13]. The most abundant organic Se is SeMet in wheat and rice, while the main Se species in Indian mustard roots are dimethyl selenide (DMeSe) and SeMeCys. However, SeCys_2_ and SeMet combine with the protein fraction, affecting the normal structure of the protein and leading to toxicity [20, 34]. Therefore, the study of the proportion of Se species and subcellular fractions in plants is required to assess Se biofortification.

The objectives of this hydroponic study were to investigate (1) the interactive effects of P combined with three types of Se fertilizer on the absorption, transport and distribution of Se in wheat and (2) the subcellular fraction and speciation of Se in response to P combined with Se treatment. Our findings will improve our understanding of the interaction of P and Se in plants and our ability to more effectively regulate the nutritional quality of winter wheat grain via the application of P and Se fertilizers in agricultural practice.

## Results

### P concentration and accumulation in wheat

Se and P application the interaction between Se and P had significant effects on P concentration and accumulation in shoots and roots, except for the P concentration and accumulation in roots influenced by Se fertilizers (*p* < 0.01; Table S1).

**Fig. 1:**
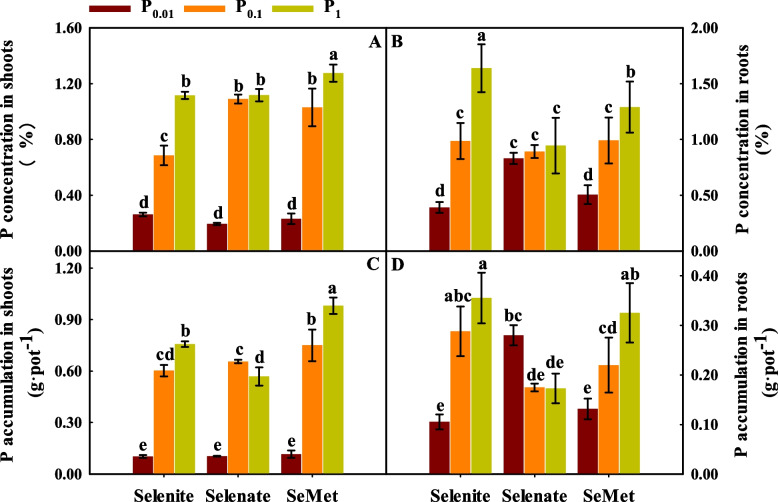
P concentration and accumulation in shoots (**A** and **C**, respectively) and roots (**B** and **D**, respectively) of winter wheat (Triticum aestivum cv. Bainong 207) seedlings as an effect of P and Se application

Compared with P_0.01_, P_0.1_ and P_1_ significantly increased the P concentration and accumulation in shoots and roots with selenite and SeMet treatment (Fig. 1). With selenate treatment, P_0.1_ and P_1_ significantly increased the P concentration and accumulation in shoots but decreased the P accumulation in roots compared to P_0.01_.

For P_0.01_, the root P concentration and accumulation with selenate treatment were higher than those with selenite and SeMet treatment (Fig. 1). For P_0.1_, the P concentrations in shoots with SeMet and selenate treatment were higher than those with selenite treatment, but the P accumulation in roots showed the opposite result. For P_1_, the highest shoot P concentration and accumulation were obtained with SeMet treatment, but the highest root P concentration and accumulation were obtained with selenite treatment.

### Se concentration and accumulation in wheat

Se and P application and the interaction between Se and P had significant effects on Se concentration and accumulation in shoots and roots (*p* < 0.01; Table S2).

**Fig. 2:**
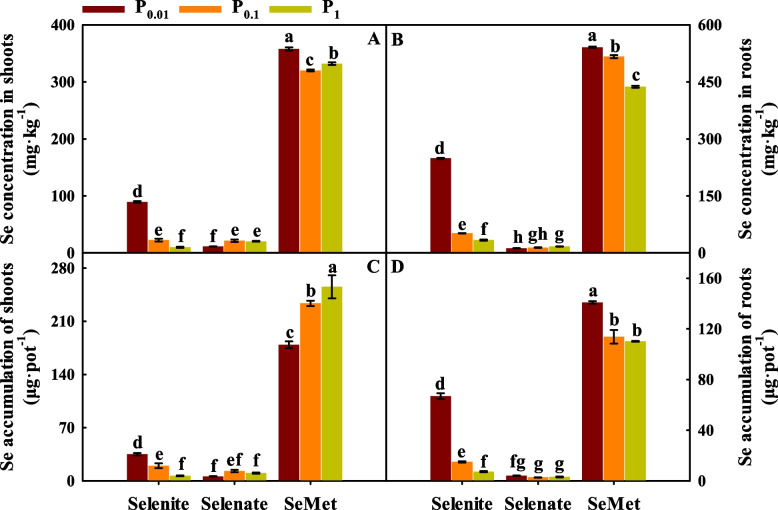
Se concentration and accumulation in shoots (**A** and **C**, respectively) and roots (**B** and **D**, respectively) of winter wheat (Triticum aestivum cv. Bainong 207) seedlings as an effect of P and Se application

With selenite treatment, the Se concentration and accumulation in shoots and roots significantly decreased with increased P application (Fig. 2). Compared with P_0.01_, P_0.1_ and P_1_ increased the Se concentration in shoots and roots with selenate treatment. With SeMet treatment, P_0.1_ and P_1_ significantly decreased the shoot and root Se concentrations and the root Se accumulation but significantly increased the shoot Se accumulation.

For each P application rate, Se concentrations and accumulation in shoots and roots with SeMet treatment were significantly higher than those with selenite and selenate treatment (Fig. 2).

### P and Se translocation and distribution in wheat

Se and P application and the interaction between Se and P had significant effects on the P and Se migration coefficients, except for the P migration coefficient influenced by P application (*p* < 0.01; Table S3).

**Fig. 3:**
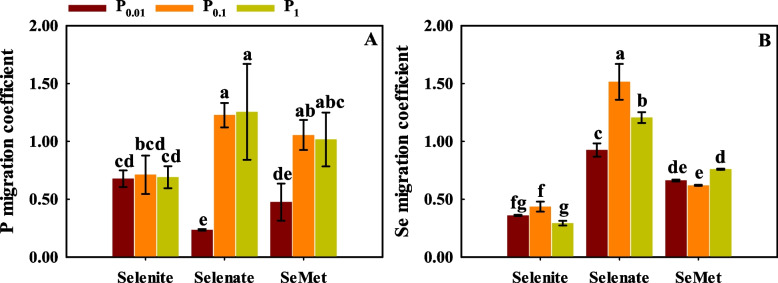
P (**A**) and Se (**B**) migration coefficient from root to shoot in tissues of winter wheat (Triticum aestivum cv. Bainong 207) seedlings as an effect of P and Se application

With selenate and SeMet treatment, P_0.1_ and P_1_ significantly increased the P migration coefficient compared to P_0.01_ (Fig. 3A). Compared with P_0.01_, P_0.1_ and P_1_ significantly increased the Se migration coefficient with selenate treatment (Fig. 3B). For P_0.01_, the P migration coefficient with selenate treatment was lower than that with selenite and SeMet treatment, but for P_0.1_ and P_1_, the P migration coefficient had the opposite result (Fig. 3A). For each P application rate, the Se migration coefficient with selenate treatment was significantly higher than that with selenite and SeMet treatment (Fig. 3B).

**Fig. 4:**
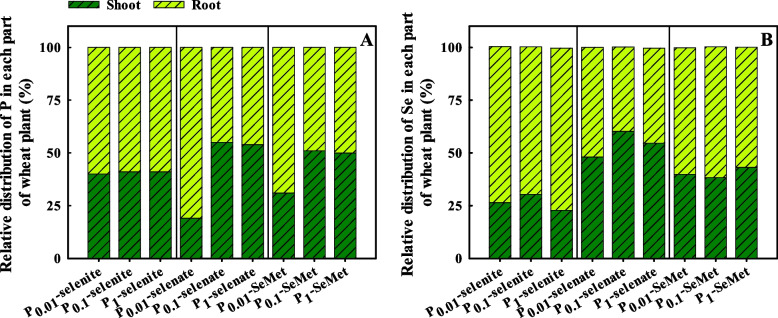
Relative distribution of P (**A**) and Se (**B**) of winter wheat (Triticum aestivum cv. Bainong 207) seedlings as an effect of P and Se application

With selenate and SeMet treatment, P_0.1_ and P_1_ significantly increased the distribution of P in shoots compared to P_0.01_ (Fig. 4A). For P_0.01_, the distribution of P in shoots with selenite treatment was higher than with selenate and SeMet treatment, but the opposite result was observed for P_0.1_ and P_1_. With selenite and selenate treatment, the distribution of Se in shoots first increased and then decreased with increasing P application (Fig. 4B). For each P application rate, the distribution of Se in shoots with selenate treatment was higher than that with selenite and SeMet treatment.

### Se subcellular fraction and distribution in wheat

Se and P application and the interaction between Se and P had significant effects on the subcellular fraction of Se in shoots and roots (*p* < 0.01; Table S4).

With selenite treatment, the Se concentration in each fraction of the three tissues all showed a significant decrease with increasing P application rates (Table 1). However, there were no pronounced differences in the Se concentrations in each fraction of shoots and roots between different P application rates with selenate treatment. With SeMet treatment, P_0.1_ and P_1_ significantly decreased Se concentrations in shoot cell organelles and in root cell walls.

**Table 1:** Subcellular fractions of Se in tissues of winter wheat (*Triticum aestivum* cv. Bainong 207) seedlings as an effect of P and Se application

Treatment		Cell wall	Cell organelle	Soluble fraction	Cell wall	Cell organelle	Soluble fraction
Se species (2 μmol·L^−1^)	P (mmol·L^−1^)	Shoot			Root		
Selenite	0.01	8.27 ± 0.08b	0.62 ± 0.04c	4.99 ± 0.74c	9.46 ± 0.32d	2.30 ± 0.27b	3.50 ± 0.23b
	0.1	2.62 ± 0.06c	0.21 ± 0.02d	0.68 ± 0.02ef	0.85 ± 0.03f	0.27 ± 0.02de	0.46 ± 0.08c
	1	0.56 ± 0.09d	0.08 ± 0.01d	0.06 ± 0.00f	0.02 ± 0.00f	0.02 ± 0.00e	0.06 ± 0.04d
Selenate	0.01	1.80 ± 0.05cd	0.31c ± 0.04cd	1.32 ± 0.03d	2.67 ± 0.11e	0.59 ± 0.07cd	0.46 ± 0.05ef
	0.1	1.77 ± 0.05cd	0.31 ± 0.03cd	1.54 ± 0.03d	2.47 ± 0.18e	0.57 ± 0.05cd	0.60 ± 0.05e
	1	1.81 ± 0.17cd	0.30 ± 0.02cd	1.55 ± 0.20d	2.46 ± 0.07e	0.47 ± 0.03cd	0.65 ± 0.03e
SeMet	0.01	28.3 ± 1.20a	8.45 ± 0.06a	8.65 ± 0.32a	31.7 ± 0.95a	2.84 ± 0.17a	4.73 ± 0.26b
	0.1	27.3 ± 0.84a	7.06 ± 0.48b	7.96 ± 0.51ab	19.5 ± 0.89b	2.78 ± 0.24a	4.61 ± 0.30b
	1	26.9 ± 1.85a	7.10 ± 0.14b	7.40 ± 0.23b	15.9 ± 1.39c	2.73 ± 0.15a	3.56 ± 0.07c

Values are the means of three independent replicates (± sd). For each trait, values followed by different letters are significantly different from each other according to two-way ANOVA followed by least significant difference (LSD) multiple comparison (*p* < 0.05)

**Fig. 5:**
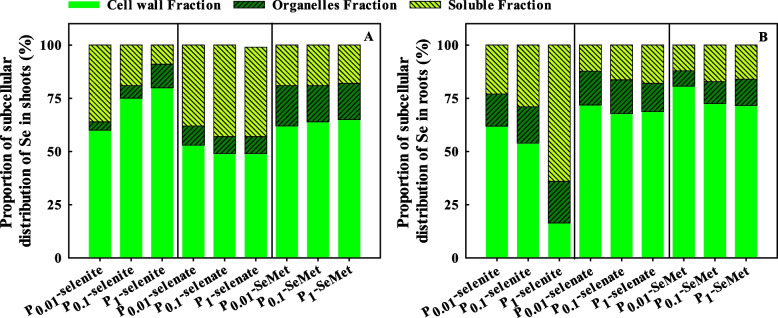
Proportion of subcellular distribution of Se in shoots (**A**) and roots (**B**) of winter wheat (Triticum aestivum cv. Bainong 207) seedlings as an effect of P and Se application

For all the treatments, the proportion of Se in the cell wall of shoots and roots was higher than that in the cell organelle and soluble fractions, except for that for P_1_-selenite (Fig. 5). With selenite treatment, the Se proportion in the cell wall of shoots increased, but the Se proportion in the soluble fractions of shoots decreased with increasing P application rates (Fig. 5A); however, P_0.1_ and P_1_ decreased the Se proportion in the cell wall of roots but increased the Se proportion in the soluble fractions of roots compared to P_0.01_ (Fig. 5B). With selenate and SeMet treatment, an increase in the P application rate had no significant influence on the proportion of Se. For each P application rate, the Se proportion in the cell wall of shoots with selenite treatment was higher than that with selenate and SeMet treatment (Fig. 5A); the Se proportion in the soluble fraction of roots with selenite treatment was higher than that with selenate and SeMet treatment, but the opposite result was observed for the Se proportion in the cell wall of roots (Fig. 5B).

### Se species

Se application had significant effects on Se (IV), Se (IV), SeCys_2_, MeSeCys and SeMet concentrations in shoots and roots (*P* < 0.01; Table S5); P application had significant effects on Se (IV), MeSeCys and SeMet concentrations in shoots as well as Se (IV), SeCys_2_, MeSeCys and SeMet concentrations in roots (*P* < 0.01); the interaction between Se and P had significant effects on Se (IV), Se (IV), MeSeCys and SeMet concentrations in shoots as well as Se (IV), Se (IV), SeCys_2_, MeSeCys and SeMet concentrations in roots (*p* < 0.01 or *p* < 0.05).

With selenite treatment, Se (IV) was detected only in roots, and increasing P application rates significantly reduced the concentrations of SeCys_2_, MeSeCys and SeMet in shoots and roots as well as Se (IV) concentrations in roots (Table 2). With selenate treatment, P_0.1_ and P_1_ decreased Se (IV) concentrations in shoots but increased those in roots compared to P_0.01_. With SeMet treatment, P_0.1_ and P_1_ significantly decreased the shoot and root SeMet concentrations but significantly increased the root SeCys_2_ concentrations.

**Table 2:** Se species in tissues of winter wheat (*Triticum aestivum cv.* Bainong 207) seedlings as an effect of P and Se application

Treatment		Se (IV)	Se (VI)	SeCys_2_	MeSecys	SeMet
Se species (2 μmol·L^−1^)	P (mmol·L^−1^)	Shoot				
selenite	0.01	ND	ND	1.36 ± 0.08b	3.59 ± 0.33b	25.6 ± 1.06d
	0.1	ND	ND	0.78 ± 0.03bc	1.41 ± 0.42c	7.31 ± 0.22e
	1	ND	ND	0.21 ± 0.05c	0.44 ± 0.10c	3.28 ± 0.38f
selenate	0.01	ND	3.44 ± 0.37a	0.54 ± 0.03c	0.42 ± 0.04c	3.47 ± 0.40f
	0.1	ND	4.12 ± 0.42a	0.55 ± 0.03c	0.67 ± 0.25c	3.35 ± 0.56f
	1	ND	0.09 ± 0.01f	0.51 ± 0.12c	0.79 ± 0.46c	3.57 ± 0.12f
SeMet	0.01	1.23 ± 0.11a	ND	3.33 ± 0.82a	13.2 ± 0.45a	90.6 ± 1.45a
	0.1	1.15 ± 0.11a	ND	3.26 ± 0.30a	12.7 ± 0.79a	54.4 ± 0.01b
	1	1.42 ± 0.12a	ND	3.71 ± 0.57a	12.5 ± 1.23a	41.4 ± 0.13c
Se species (2 μmol·L^−1^)	P (mmol·L^−1^)	Root				
selenite	0.01	1.69 ± 0.05bc	ND	10.9 ± 0.68a	3.08 ± 0.34d	16.3 ± 0.31d
	0.1	0.71 ± 0.03cd	ND	1.85 ± 0.16c	1.13 ± 0.10de	6.30 ± 0.49e
	1	0.21 ± 0.01d	ND	0.58 ± 0.01d	0.66 ± 0.02e	3.15 ± 0.18ef
selenate	0.01	ND	0.64 ± 0.10b	ND	0.79 ± 0.04de	1.64 ± 0.09f
	0.1	ND	1.04 ± 0.13b	ND	1.04 ± 0.05de	1.23 ± 0.09f
	1	ND	2.14 ± 0.46a	ND	2.01 ± 0.10cd	1.19 ± 0.11f
SeMet	0.01	2.54 ± 0.18ab	ND	1.90 ± 0.07cd	25.3 ± 1.14a	65.3 ± 3.40a
	0.1	2.72 ± 0.72ab	ND	6.29 ± 0.39b	17.2 ± 0.85b	52.5 ± 0.90b
	1	3.49 ± 0.78a	ND	10.5 ± 0.83a	16.8 ± 0.78b	38.3 ± 4.30cd

Values are the means of three independent replicates (± sd). For each trait, values followed by different letters are significantly different from each other according to two-way ANOVA followed by least significant difference (LSD) multiple comparison (*p* < 0.05). ND indicates that the value was below the detection limit. Se (IV), selenite; Se (VI), selenate; SeCys_2_, selenocystine; MeSeCys, Se-methyl-selenocysteine; SeMet, selenomethionine

**Fig. 6:**
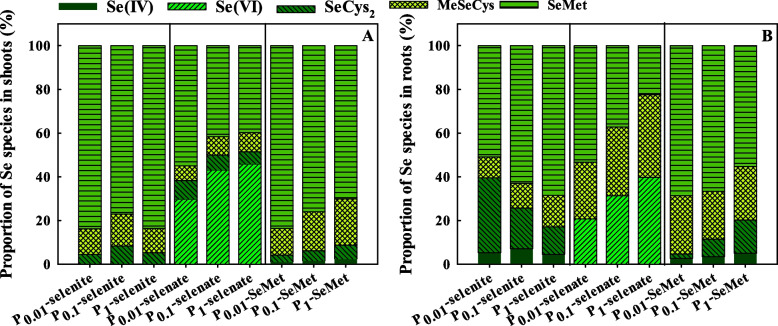
Proportion of Se species in shoots (**A**) and roots (**B**) of winter wheat (Triticum aestivum cv. Bainong 207) seedlings as an effect of P and Se application. Se (IV), selenite; Se (VI), selenate; SeCys_2_, selenocystine; MeSeCys, Se-methyl-selenocysteine; SeMet, selenomethionine

The most abundant Se species were SeMet and MeSeCys in most cases (Fig. 6). With selenite treatment, the proportion of MeSeCys and SeMet in roots gradually increased, but the SeCys_2_ proportion in roots decreased with increasing P application rates. With selenate treatment, an increase in P application increased the Se (VI) and MeSeCys proportions in each tissue but decreased the shoot SeMet proportion. With SeMet treatment, the SeMet proportion in shoots and roots was reduced, but the SeCys_2_ proportion in each tissue increased with increasing P application rate.

### Dry matter weights

Se and P application and the interaction between Se and P had significant effects on the dry matter weight in the shoots and roots of winter wheat (*P* < 0.01; Table S6).

Compared with P_0.01_, P_0.1_ and P_1_ significantly increased the dry matter weights of shoots under selenite and SeMet treatments (Table 3). Both P application rates (P_0.1_ and P_1_) decreased the dry matter weights of roots in the selenate and SeMet treatments, respectively; however, in the presence of selenite, the dry matter weights of roots in the P_0.1_ treatment were higher than those in the P_0.01_ and P_1_ treatments.

**Table 3:** Shoot and root dry matter weights of winter wheat (*Triticum aestivum* cv. Bainong 207) seedlings as an effect of P and Se application

Treatment		Shoot/(g, DW)	Root/(g, DW)
Se species (2 μmol·L^−1^)	P (mmol·L^−1^)		
Selenite	0.01	0.39 ± 0.01f	0.27 ± 0.01bc
	0.1	0.88 ± 0.05a	0.29 ± 0.01b
	1	0.68 ± 0.00bc	0.22 ± 0.01d
Selenate	0.01	0.53 ± 0.02de	0.34 ± 0.01a
	0.1	0.60 ± 0.01cd	0.20 ± 0.01de
	1	0.51 ± 0.02e	0.19 ± 0.02e
SeMet	0.01	0.50 ± 0.01e	0.26 ± 0.00bc
	0.1	0.73 ± 0.01b	0.22 ± 0.01de
	1	0.77 ± 0.03b	0.25 ± 0.00cd

Values are the means of three independent replicates (± sd). For each trait, values followed by different letters are significantly different from each other according to two-way ANOVA followed by least significant difference (LSD) multiple comparison (*p* < 0.05)

As P was applied at 0.01 mmol L^−1^, the dry matter weight of shoots and roots in the selenate treatment was higher than that in the selenite and SeMet treatments; at P_0.1_, the dry matter weight of shoots and roots showed selenite > SeMet > selenate treatment; however, at P_1_, the dry matter weight of shoots and roots in the selenite and SeMet treatments was significantly higher than that in the selenate treatment.

### Root morphology parameters

Se and P application and the interaction between Se and P had significant effects on the root length, surface, volume, tip number and forks, except for the average root diameter influenced by P application (*p* < 0.01 or *p* < 0.05; Table S7).

With selenite treatment, the total root length, surface area, tip number and fork first increased and then decreased as P application increased, but P_0.1_ and P_1_ decreased the root volume and average diameter (Table 4). The root length, surface area, volume, tip number and fork significantly decreased with increasing P application rates with selenate treatment. With SeMet treatment, the total root length, surface area, volume, average diameter and fork first increased and then decreased with increasing P application, but P_0.1_ and P_1_ significantly increased the root tip numbers.

**Table 4:** Root morphology parameters of winter wheat (Triticum aestivum cv. Bainong 207) seedlings as an effect of P and Se application

Treatment		Total root length (mm)	Surface area (cm^2^)	Volume (cm^3^)	Average diameter (mm)	Tip numbers	Forks
Se species (2 μmol·L^−1^)	P (mmol·L^−1^)						
Selenite	0.01	782 ± 4.55c	66.9 ± 0.37c	0.44 ± 0.02b	0.27 ± 0.01b	976 ± 5.79d	1810 ± 0.41g
	0.1	993 ± 9.04a	84.4 ± 3.02a	0.42 ± 0.00b	0.23 ± 0.00f	1856 ± 3.67a	3486 ± 4.90a
	1	725 ± 5.89e	54.8 ± 1.22f	0.33 ± 0.01e	0.24 ± 0.00de	1448 ± 7.79b	2523 ± 9.09d
Selenate	0.01	895 ± 4.53b	71.0 ± 2.39b	0.42 ± 0.01b	0.24 ± 0.00de	1414 ± 7.41b	2887 ± 5.72b
	0.1	791 ± 5.62c	59.0 ± 1.17e	0.37 ± 0.02cd	0.24 ± 0.01de	1210 ± 4.99c	2363 ± 6.94e
	1	645 ± 4.02g	53.0 ± 2.22f	0.35 ± 0.02de	0.26 ± 0.00bc	1020 ± 6.94d	1983 ± 12.4f
SeMet	0.01	764 ± 8.76d	62.5 ± 0.73d	0.39 ± 0.01c	0.26 ± 0.00bc	750 ± 2.83f	1949 ± 7.76g
	0.1	887 ± 6.88b	70.7 ± 0.06b	0.54 ± 0.01a	0.30 ± 0.01a	830 ± 4.90e	2748 ± 7.41c
	1	671 ± 5.62f	54.4 ± 0.73f	0.35 ± 0.01de	0.25 ± 0.00cd	1243 ± 8.83c	1686 ± 6.94i

Values are the means of three independent replicates (± sd). For each trait, values followed by different letters are significantly different from each other according to two-way ANOVA followed by least significant difference (LSD) multiple comparison (*p* < 0.05)

For P_0.01_, the total root length, surface area, tip number and forks with selenate treatment were higher than those with selenite and SeMet treatment (Table 4). For P_0.1_ and P_1_, the total root length, tip number and forks with selenite treatment were higher than those with selenate and SeMet treatment; however, for P_0.1_, the root volume and average diameter with SeMet treatment were higher than those with selenite and selenate treatment.

### Photosynthesis

Se and P treatments and the interaction between Se and P had significant effect on net photosynthetic rate (*P*_*n*_), stomatal conductance (*G*_*s*_), intercellular CO_2_ concentration (*C*_*i*_) and transpiration rate (*T*_*r*_) (*p* < 0.01; Table S8).

Compared with P_0.01_, P_0.1_ and P_1_ significantly increased *P*_*n*_, *G*_*s*_, *C*_*i*_ and *T*_*r*_ with each Se fertilizer, with the highest values for P_0.1_ with both selenite and SeMet (Table 5).

**Table 5:** Photosynthetic characteristics of winter wheat (*Triticum aestivum* cv. Bainong 207) seedlings as an effect of P and Se application

Treatment		Net photosynthetic rate (μmol CO_2_ m^−2^ s^−1^)	Stomatal conductance (mol H_2_O m^−2^ s^−1^)	Intercellular CO_2_ concentration (μmol CO_2_ m^−2^)	Transpiration rate (mol H_2_O ^−2^ s^−1^)
Se species (2 μmol·L^−1^)	P (mmol·L^−1^)				
Selenite	0.01	7.73 ± 0.36d	0.03 ± 0.01f	65.2 ± 4.56f	0.45 ± 0.10e
	0.1	13.4 ± 0.16a	0.13 ± 0.00c	381 ± 10.2b	2.13 ± 0.08c
	1	11.0 ± 0.65c	0.09 ± 0.00e	331 ± 7.17e	1.34 ± 0.10d
Selenate	0.01	8.54 ± 0.36d	0.08 ± 0.00e	355 ± 10.5d	1.31 ± 0.07d
	0.1	10.4 ± 0.37c	0.12 ± 0.00d	383 ± 9.81b	1.87 ± 0.06c
	1	10.0 ± 0.71c	0.15 ± 0.01c	383 ± 6.90b	2.00 ± 0.29c
SeMet	0.01	7.61 ± 0.40d	0.04 ± 0.01f	365 ± 10.8cd	0.58 ± 0.11e
	0.1	12.0 ± 0.15b	0.22 ± 0.01a	407 ± 3.48a	3.22 ± 0.18a
	1	10.7 ± 0.33c	0.20 ± 0.00b	403 ± 2.08a	2.81 ± 0.04b

Values are the means of three independent replicates (± sd). For each trait, values followed by different letters are significantly different from each other according to two-way ANOVA followed by least significant difference (LSD) multiple comparison (*p* < 0.05)

For P_0.01_, *G*_*s*_ and *T*_*r*_ with selenate treatment were significantly higher than those with selenite and SeMet treatment, and *Ci* with selenate and SeMet treatment was significantly higher than that with selenite treatment (Table 5). For P_0.1_ and P_1_, *G*_*s*_, *C*_*i*_ and *T*_*r*_ with SeMet treatment were significantly higher than those with selenite and selenate treatment, but *P*_*n*_ with selenite treatment was significantly higher than that with selenate and SeMet treatment for P_0.1_.

## Discussion

### P and Se uptake, translocation and distribution

The mechanisms of uptake and transport of selenite, selenate and SeMet are different in plants [11, 35]. Selenite, selenate and SeMet are absorbed in plants via P transporters, sulfur (S) transporters and aquaporins, respectively [17, 23, 36–38]. In this study, at the same P application level, the Se concentration of each wheat organ with SeMet treatment was higher than that with selenite and selenate treatment (Fig. 2), which is in agreement with the results of Ali et al. [39], Huang et al. [40] and Eich-Greatorex et al. [41], who pointed out that the uptake rate of SeMet in rice roots was significantly higher than that of inorganic Se based on Se concentration-dependent kinetics. This might be related to the fact that organic selenium has higher biological activity and absorption efficiency than inorganic selenium [42]. Some studies pointed that foliar spraying of selenite had higher Se concentration than selenate due to selenite can rapidly convert to organic forms [21, 43]. However, other studies showed that the Se concentration in each tissue of wheat with selenate treatment was higher than that with selenite treatment because selenate had greater bioavailability than selenite [30, 44, 45]. Wang et al. [33] noted that the Se concentration of tomato relative to Se application rate, when Se application rate was 0.0175-0.2998 mg·L^−1^, selenate treatment was tenfold greater than that with selenate treatment in hydroponic experiments, but the opposite result was observed in other Se concentrations. Luo et al. [46] demonstrated that mycorrhizal inoculation improved the valid absorption area of roots and promoted the increased uptake of Se. In our study, Se concentration of shoots and roots with selenite treatment was higher than that with selenate treatment (Fig. 2A and B), this result might be attributed to an appropriate amount of P combined with selenite treatment altered root morphological parameters compared to that with selenate treatment (Table 4). The current results suggested that the difference in Se concentration between selenite, selenate and SeMet can be explained by their different uptake mechanisms, Se application level and Se application methods [11, 46, 47].

Previous studies have shown that Se absorption and accumulation in plants are affected by P application [11, 45]. In our study, an increase in the P application rate significantly reduced Se concentration and accumulation in shoots and roots with selenite treatment (Fig. 2). Zhang et al. [23] and Li et al. [38] demonstrated that increasing the P application rate significantly decreased the expression of P transporters in roots and further decreased Se concentrations in plants. Liu et al. [48] also noted that P_3.1_ and P_31_ significantly inhibited Se absorption and decreased the Se concentration of wheat compared with P_0.31_, which is consistent with our results, possibly because P application inhibited the uptake of selenite. With selenate treatment, P_0.1_ and P_1_ increased the Se concentration in shoots and roots (Fig. 2A and B). Previous studies demonstrated that selenate uptake by plants via sulfate (S) transporters due to the similar chemical properties between selenate and sulfate [49, 50]. Schmittgen et al. [51] indicated that -S significantly upregulated the expression of *Sultr1;1* compared to +S treatment and increased Se accumulation in grain. However, Zhang et al. [30] noted that P fertilizer activated organic matter-bound Se and increased the Se concentration and accumulation of wheat with selenate treatment, but significantly inhibited Se uptake with selenite treatment due to antagonism between the absorption of P and Se [45], this is consistent with the results of our study. Previous studies showed that SeMet are absorbed in plants be found to be an energy-dependent symport process involving H^+^ transport [25, 52]. The present results showed that an increase in P application rates decreased the Se concentration in shoots and roots with SeMet treatment (Fig. 2A and B), which might be related to the dilution effect by an increase in plant growth as a result of increased P application rates or P involved in energy metabolism process. Therefore, the effect of P application on Se concentration and accumulation depended on the type of Se fertilizer, but further verification of this possibility is still needed [53].

The translocation factor is used to characterize the transfer ability of nutrient elements in plants [54]. Previous studies have shown that the migration of Se from roots to shoots is closely related to the type of Se [55]. Selenate has a stronger mobility, but a small amount of selenite is transported in the xylem of plants [56, 57]. The Se migration coefficient with selenate treatment was 40-90 % higher than that with selenite treatment [39], selenite readily accumulates in roots, selenate is more easily transported from root to shoot, and 70 % of the total Se is present in the straw of rice [58–60]. The present results showed that the Se migration coefficient with selenate treatment was significantly higher than that with selenite and SeMet treatment at the three P application rates (Fig. 3B), resulting in most Se being distributed in the roots with selenite treatment, while more Se was found in the shoots with selenate treatment (Fig. 4B), which is in agreement with the findings of Wang et al. [22]. Additionally, a high P application rate (P_1_) reduced the Se migration coefficient with selenite treatment (Fig. 3B). Zhang et al. [23] showed that P transporters (*OsPT6*) are involved in Se migration from roots to shoots in rice, and P application decreased the Se translocation coefficient. Lazard et al. [61] also demonstrated that there is competition between P and selenite ions for both Pi transport systems by studying the corresponding kinetic parameters. In contrast, the migration coefficient and distribution of P and Se in shoots increased with increasing P application rates with selenate treatment (Figs. 3 and 4), which suggested that P and selenate had synergistic effects on the uptake of P and Se in plants. This might be because P application enhanced transpiration and further promoted selenate translocation in plants [62].

### Subcellular distribution of Se

The compartmentalization effect of metals and metal-like elements in cells can greatly affect the level of free heavy metal ions in cells, thus affecting the movement of ions in plants [63, 64]. Se can be considered both a nutrient and toxic to plants because the gap between beneficial and toxic levels is narrow [65, 66]. Roots have a known series of important functions for defending against toxic elements, including binding elements to cell walls or sequestering them in vacuoles, and then inhibiting the translocation of these elements to shoots [67, 68]. In our study, the subcellular distribution of Se in each part of wheat occurred in the order of cell wall > cell soluble fraction > cell organelles with the three Se fertilizers, regardless of P application (Fig. 5B). Su et al. [69] found that the cell wall and vacuole (soluble fraction) can sequester more metal ions to limit the movement of ions in plants. A previous study demonstrated that the cell wall plays an important role in Se binding [67]. In the wheat roots, an increase in P application decreased the Se concentration in each fraction and the distribution of Se in the root cell wall but increased that in the root soluble fraction with selenite treatment (Table 1 and Fig. 5B). Our results are consistent with the findings of Wang et al. [13], who noted that compared with P-normal treatment, P deficiency combined with -P+Se treatment increased the proportion of Se distributed in the root cell wall but reduced the Se distribution in the root soluble fraction. These results indicated that increasing the P supply could inhibited the transmembrane transportation of Se to reduce cell wall-bound Se and then decreased the Se migration coefficient from roots to shoots in wheat [70]. In contrast, an increase in P supply level increased the distribution of Se in the shoot cell wall and cell organelles and decreased the shoot soluble fraction, which is consistent with the results of Liu et al. [48]. Winkel et al. [71] indicated that increasing distribution of Se in the cell organelle is related to enhance metabolism of Se. Therefore, this result may also indicate that an increase in the P supply level may promoted Se metabolism. However, P application had no obvious influence on the subcellular distribution of Se with SeMet and selenate treatment (Table 1, and Fig. 5), which might be because the uptake pathway of selenate is via sulfate transporters and that of SeMet is via aquaporins, respectively, but not phosphate transporters [17]. Li et al. [11] also indicated that P application significantly inhibited the influx of selenite, and had no significant effect on Se uptake with selenate treatment, whereas S-starved treatment increased Se uptake and translocation by 9.5-fold in the presence of selenate.

### Se species

It is well known that Se is chemically similar to S [72], and Se is converted into organic forms through the S metabolic pathway after being absorbed by plant roots [73, 74]. Selenate is first converted into selenite via two enzymes known as ATP sulfurylase (APS) and APS reductase (APR) in plants [75], and then selenite is converted into selenide and SeCys by sulfite reductase (SiR) and cysteine synthase; some SeCys can be converted into SeMet by a series of enzymes [66, 76]. In our study, the main Se species in wheat was SeMet (Table 2, Fig. 6), which is consistent with the results of previous studies [77, 78]. However, different Se fertilizers had significant effects on Se species in plants. For example, SeMeCys was dominant when selenite was supplied, but Se (VI) was dominant with selenate addition in *Brassica rapa* [79]. SeMet was the major form of Se in the roots and shoots of rice with SeNPs or selenite treatment [80]. Our study showed that Se(VI) (20.8-45.8 %) and SeMet (22.5-55.0 %) were the main species in the roots and shoots of wheat when selenate was applied (Fig. 6), which is consistent with the results of Li et al. [11] and Li et al. [81]. This might be related to the fact that selenate is not completely converted into organic Se as a result of the high uptake rates or storage of Se in vacuoles [20, 82]. Selenite was quickly metabolized to organic Se (SeCys_2_, MeSeCys and SeMet) after absorption in roots and then transported to shoots in the form of organic Se [37], which might be the reason that Se (IV) was detected only in roots (Table 2). Oliveria et al. [83] also showed that inorganic Se was most likely transformed in root chloroplasts because selenoamino acids are known to exist in the chloroplasts of plant roots by genetic control. With SeMet treatment, SeMet and MeSeCys accounted for more than 79.0 % of the total Se, but low concentrations of Se (IV) and SeCys_2_ were detected in roots and shoots (Fig. 6). This is consistent with the results of Wang et al. [52], who showed that SeMet and MeSeCys were the dominant species in organic Se-treated wheat.

In the present study, increasing the P application rate promoted the transformation of Se species with three types of Se fertilizers (Table 2, Fig. 6). With selenite treatment, an increase in P application elevated the proportion of SeMet, but decreased the proportion of SeCys_2_ (Fig. 6B), this result was consistent with the result of Wang et al. [13], who showed that additional P under P-deficient condition induced a strong reduction in the proportion of SeMet but increased the proportion of Se (VI) and SeCys_2_ in the roots of rice. Moreover, the proportion of Se (VI) was increased, but the proportion of SeMet in shoots and roots was decreased by P application with selenate treatment (Fig. 6). A previous study suggested that little selenate was assimilated into organic forms due to selenate was highly mobile in xylem transport [11]. Therefore, it was speculated that an increase in P application increased Se migration coefficient with selenate treatment due to selenate can rapidly mobile (Fig. 3B). However, with SeMet treatment, increasing the P supply decreased the proportion of SeMet but increased the proportion of SeCys_2_ in roots and shoots (Fig. 6). SeCys_2_ can be converted into MeSeCys via methylation and SeMet by a series of enzymes (i.e., cystathionine-γ -synthase, cystathionine-β-lyase, and Met synthase) [76], and P plays an important roles on protein synthesis and energy metabolism. Our study showed that P application had different influences on the proportion of Se species in each organ with three types of Se sources, which was probably due to their different uptake mechanisms, and altered the bioavailability of Se [13, 17]. However, further studies on how Se metabolism is mediated by P application are still needed.

### Dry matter weight, root morphological parameters and photosynthesis of wheat

Studies demonstrated that root growth and photosynthesis of plant play a vital role in the growth and yield of crops, root and photosynthesis are closely related to the uptake of nutrient elements and organic matter accumulation, respectively [84]. Studies showed that plants can adjust their root length, volume, surface area, average diameter, tip numbers and forks to adapt to different P conditions [85], and P starvation decreased ATP synthase activity because P is the substrate for ATP synthesis in the chloroplast stroma and also significantly decreased the photosynthesis of barley [86]. Our results showed that an increase in P application had significant effect on root morphological parameters and photosynthesis of winter wheat in each Se sources (Tables 4 and 5). Loudari et al. [87] noted that an adequate amount of P significantly increased the photosynthetic parameters and biomass in wheat. Although Se is not an essential element for plants, it is beneficial for plant growth and photosynthesis [88]. Se application increased photosynthetic pigments content, including chlorophyll a, chlorophyll b and carotenoid, and then enhanced photosynthesis [89]. Roda et al. [90] analysed the transcriptome of rice flag leaves and found that selenite application promoted vitamin biosynthesis and metabolism, which are involved in photosynthesis in rice. Low-dose selenite treatment (0.5 and 1 mg·kg^−1^) stimulated plant growth by enhancing root activities, but high Se levels (50 and 100 mg·kg^-1^) decreased root activity [91]. Different Se species as well as their application rates affect the crop yield and biomass of plants [33, 92, 93]. Previous studies showed that 2 mg·kg^–1^ Se (VI) significantly inhibited wheat plant growth and decreased plant height compared to 0.5 mg·kg^–1^ Se (IV) treatment [94]. Li et al. [32] pointed that selenite application can improve the root activity of lettuce and increase the root fresh weights and root length, but selenate has no significant influence on root activity. However, some studies indicated that P nutritional status significantly affects plant growth under different Se application conditions [30, 95]. In our study, P application rate had different effects on wheat growth with different Se fertilizers (Table 3). These may indicate that the effect of Se fertilizer on the growth of plant is closely related to the type of Se, Se application levels and P conditions [22].

In the present study, an increase in P application promoted plant growth and increased the dry matter weight in shoots and roots with selenite treatment, where the highest value appeared under the P_0.1_ treatment, this may be because an appropriate amount of P combined with selenite significantly promoted the uptake of nutrient elements and organic matter accumulation, and then promoted plant growth by improved photosynthesis and root morphology parameters (the total root length, surface area, tip numbers and forks) of winter wheat (Tables 4 and 5), which is in agreement with the results of Nie et al. [95], who pointed out that P application increased the biomass of shoots, and P combined with selenite promoted root growth [49]. Qin et al. [96] also noted that selenite application significantly altered root morphological parameters and photosynthesis with Cd treatment, and then remitted Cd toxicity. However, P_0.1_ and P_1_ significantly decreased the dry matter weight in roots with selenate treatment (Table 3). Previous studies indicated that P starvation caused a large number of crown roots, a high lateral root density, and a large number of adventitious roots with a shallow root growth angle, but sufficient P application had exactly the reverse effect [97, 98]. It was revealed that P is critical in promoting plant growth in agricultural practices, and selenate application had no obvious effect on the root morphological parameters when P was combined with selenate compared to that with selenite and SeMet, which may be because P plays a leading role in root growth. Therefore, increasing P application decreased the dry matter weight in roots through inhibited root morphology parameters (Tables 3 and 4). Our results also showed that photosynthesis (*P*_*n*_ and *G*_*s*_) and root morphological parameters with selenite and SeMet treatment were higher than those with selenate treatment when P was supplied at 0.1 mmol·L^-1^ (Table 5). This may be because selenite and SeMet application can increased the photosynthetic pigment and altered root activity of plants when Se application level was 2 μmol·L^-1^, but the opposite result was observed for selenate. The reason for this difference may be that the selenate application level was too high for winter wheat in our experiment, resulting in plant toxicity, decreased plant organic matter accumulation and the dry matter weight of roots (Table 3) [89, 99, 100]. Zhang et al. [30] pointed that compared with no P fertilizer treatment, P fertilizer treatment significantly increased wheat biomass with selenate treatment (1 or 2 mg·kg^-1^) in a pot experiment. The reasons for these different results may be related to the differences in Se and P application rates and cultivation methods [22, 38]. This result showed that an appropriate amount of P combined with selenite can promote winter wheat growth compared with selenate and SeMet treatment, and also pointed out that different Se sources have different responses to the dry matter weight of wheat under the same Se application level [32, 49, 101].

## Conclusion

We first reported that the effects of the uptake, translocation and subcellular distribution and species of Se in wheat by P application with three types of Se fertilizers. With selenite treatment, although increased P application decreased Se uptake and translocation, an appropriate amount of P promoted wheat growth, photosynthetic and altered the Se species fraction, and then has significantly influence on Se bioavailability in wheat. P fertilizer application should be balanced in actual production. With selenate treatment, an increase in P application promoted Se uptake and translocation, but P combined with selenate application inhibited root growth of winter wheat, it caused toxicity to plant growth when Se application level was 2 μmol·L^−1^ in this experiment. With SeMet treatment, increased P application decreased Se concentration of winter wheat, this might be related to the dilution effect by an increase in plant growth, but significantly increased photosynthesis and the dry matter weight of shoots. The proportion of Se species in winter wheat was related to the Se sources and P application rate. Therefore, we should consider rational application of P fertilization and the types of Se fertilizer in soil in field agricultural production. Our results provided critical reference on the effective agricultural production of Se-enriched wheat. However, further studies are needed to elucidate the transformation of Se species by P application.

## Materials and methods

### Plant materials and treatment

A hydroponic experiment was conducted in a greenhouse with controlled environmental conditions of approximately 14/10 h of light/dark, 22/18 °C air temperatures, approximately 500 µmol m^−2^ s^−1^ photon flux density, and 65 % relative humidity.

Winter wheat (*Triticum aestivum cv*. Bainong 207, purchased from Henan Agricultural High Tech Group Co., Ltd.) seeds were germinated in deionized water at 25 °C for four days after sterilization in 5 % (v/v) NaClO solution for 15 min. Then, uniform wheat seedlings were transplanted into a plastic container containing 2 L nutrient solutions as described previously by Arnon et al. (1940), which consisted of 945 mg L^−1^ Ca(NO_3_)_2_·4H_2_O, 607 mg·L^−1^ KNO_3_, 493 mg·L^−1^ MgSO_4_·7H_2_O, 20 mg·L^−1^ EDTA-Fe, 2.86 mg·L^−1^ H_3_BO_3_, 1.81 mg·L^−1^ MnCl_2_·4H_2_O, 0.22 mg·L^−1^ ZnSO_4_·7H_2_O, 0.08 mg·L^−1^ CuSO_4_·5H_2_O, and 0.02 mg·L^−1^ (NH_4_)_6_Mo_7_O_24_·4H_2_O.

### Experimental design

P was added to the solutions as NaH_2_PO_4_·2H_2_O at three rates: 0.01, 0.1 and 1 mmol·L^−1^. Se was added as Na_2_SeO_3_, Na_2_SeO_4_ and SeMet at one rate of 2 μmol·L^−1^. Each treatment was performed in triplicate. Seedlings were supplied with full-strength nutrient solutions until sampling, except for the quarter-strength and half-strength solutions supplied during the first and second weeks, respectively. The solution was renewed every three days to ensure a sufficient nutrient concentration. A solution of 5 % HCl was used to soak all vessels for one week, and then deionized water was used to wash those vessels more than three times. After cultivation for 21 d, the roots and shoots of 14 seedlings were separated, dried at 70 ± 5 °C, and analysed for dry weights and concentrations of P and Se. The leaves, stems, and roots of the other six seedlings were immediately frozen in liquid nitrogen and then stored at − 20 °C for further subcellular fractionation and Se speciation analysis.

### Analysis of the root morphology

Three 14-d-old seedlings in each treatment were analysed in terms of their root morphological characteristics. According to the method of Nie et al. [102], the root length, root surface area, root volume, average root diameter, root tip number and forks were measured using the root imaging analysis software WinRHI-ZO Version 2009 PRO (Regent Instruments, Quebec City, Canada).

### Analysis of the photosynthetic parameters

According to the method of Shi et al. [103], three uniform seedlings of each treatment were selected for photosynthetic parameter analysis. The net photosynthetic (*P*_*n*_), stomatal conductance (*G*_*s*_), intercellular CO_2_ concentration (*C*_*i*_) and transpiration rate (*T*_*r*_) in the leaves of 21-d-old seedlings were measured from 9:00 to 11:00, and the 3rd fully expanded leaf was selected using the LI-6400XT photosynthetic-fluorescence assay system (USA, LI-COR).

### Analysis of the P concentration of wheat

According to the method of Bao. [104], after being digested with H_2_SO_4_-HClO_4_, the vanadate-molybdate-yellow colorimetric method was used to estimate the plant phosphorus concentration.

### Analysis of the Se concentration of wheat

According to the method of Nie et al. [105], approximately 0.5 g of plant samples (shoot or root) was digested with HNO_3_-HClO_4_ (4:1) at 180 °C. The digest was then restored to volume with 6 mol·L^−1^ HCl, cooled, filtered and brought to volume with deionized water. The Se concentration was determined by hydride generation atomic fluorescence spectrometry (HG-AFS-8220, Beijing Titan Instruments Co., China).

### Subcellular fraction separation

According to the methods described by Wan et al. [63], approximately 0.4 g of frozen samples (shoot or root) was homogenized in 10 mL of extraction buffer containing 1.0 mM dithioerythritol, 250 mM sucrose, and 50 mM Tris–HCl (pH 7.5). The homogenate was centrifuged at 300 × g for 10 min, and the residue constituted the cell wall fraction (F1). The supernatant was then centrifuged at 20,000 × g for 30 min, and the precipitate and the supernatant were taken as the organelle fraction (F2) and the soluble fraction (F3), respectively. The soluble fraction was diluted to 50 mL with 5 % HNO_3_ (GR) prior to elemental determination. All steps were performed at 4 °C.

### Se speciation determination

According to the methods described by Wang et al. [106], 0.05 g of the samples (shoot or root) was placed in 15 mL centrifugal tubes with 5 mL Tris–HCl. After ultrasonication for 30 min, 50 mg cellulase and 20 mg protease K were added. The mixture was incubated in an oscillation box with horizontal shaking (250 r·min^−1^) at 50 ℃ ± 2 ℃ for 18 h. After adding 20 mg protease K, the mixture was then incubated in an oscillation box with horizontal shaking (250 r·min^−1^) at 37 ℃ ± 2 ℃ for 18 h. The hydrolysate was centrifuged at 10,000 r·min^−1^ for 30 min, and the supernatant was filtered through a 0.22 µm cellulose nitrate filter (Millipore, Billerica, MA, United States). Subsequently, the filtrate was stored at -80 °C for Se speciation analysis using high-performance liquid chromatography-ultraviolet treatment-hydride generation-atomic fluorescence spectrometry (HPLC–UV-HG-AFS; SA-50, Ji tian Instruments Co., Beijing, China).

### Data analysis

All data were statistically analysed by two-way ANOVA with LSD multiple comparison at a 5% level (*p* < 0.05) using SPSS 18.0.

## Supplementary Information

**Additional file 1: Table S1.** Two-way analysis of variance (ANOVA) of the effects of Se, P treatment as well as their interactions on the P concentration and accumulation of winter wheat (*Triticum aestivum* cv Bainong 207) grown under greenhouse conditions. **Table S2.** Two-way analysis of variance (ANOVA) of the effects of Se, P treatment as well as their interactions on the Se concentration and accumulation of winter wheat (*Triticum aestivum* cv Bainong 207) grown under greenhouse conditions.** Table S3.** Two-way analysis of variance (ANOVA) of the effects of Se, P treatment as well as their interactions on the P and Se migration coefficient of winter wheat (*Triticum aestivum* cv Bainong 207) grown under greenhouse conditions. **Table S4.** Two-way analysis of variance (ANOVA) of the effects of Se, P treatment as well as their interactions on the subcellular fractions of Se in tissues of winter wheat (*Triticum aestivum* cv Bainong 207) grown under greenhouse conditions. **Table S5.** Two-way analysis of variance (ANOVA) of the effects of Se, P treatment as well as their interactions on Se species in tissues of winter wheat (*Triticum aestivum* cv Bainong 207) grown under greenhouse conditions. **Table S6.** Two-way analysis of variance (ANOVA) of the effects of Se, P treatment as well as their interactions on the dry matter weight of winter wheat (*Triticum aestivum* cv Bainong 207) grown under greenhouse conditions.** Table S7.** Two-way analysis of variance (ANOVA) of the effects of Se, P treatment as well as their interactions on the root morphology of winter wheat (*Triticum aestivum* cv Bainong 207) grown under greenhouse conditions.** Table S8.** Two-way analysis of variance (ANOVA) of the effects of Se, P treatment as well as their interactions on the photosynthetic parameter of winter wheat (*Triticum aestivum* cv Bainong 207) grown under greenhouse conditions.

## Data Availability

The datasets generated or analysed during the current study are available from the corresponding author upon reasonable request.
