# Supplementary material for: Selenium uptake, translocation, subcellular distribution and speciation in winter wheat in response to phosphorus application combined with three types of selenium fertilizer
Source: BMC Plant Biol. 2023 Apr 27;23:224. doi: 10.1186/s12870-023-04227-6 (PMC10134582; doi:10.1186/s12870-023-04227-6)
Supplement: Supplementary file 1 — Additional file 1: Table S1. Two-way analysis of variance (ANOVA) of the effects of Se, P treatment as well as their interactions on the P concentration and accumulation of winter wheat (Triticum aestivum cv Bainong 207) grown under greenhouse conditions. Table S2. Two-way analysis of variance (ANOVA) of the effects of Se, P treatment as well as their interactions on the Se concentration and accumulation of winter wheat (Triticum aestivum cv Bainong 207) grown under greenhouse conditions. Table S3. Two-way analysis of variance (ANOVA) of the effects of Se, P treatment as well as their interactions on the P and Se migration coefficient of winter wheat (Triticum aestivum cv Bainong 207) grown under greenhouse conditions. Table S4. Two-way analysis of variance (ANOVA) of the effects of Se, P treatment as well as their interactions on the subcellular fractions of Se in tissues of winter wheat (Triticum aestivum cv Bainong 207) grown under greenhouse conditions. Table S5. Two-way analysis of variance (ANOVA) of the effects of Se, P treatment as well as their interactions on Se species in tissues of winter wheat (Triticum aestivum cv Bainong 207) grown under greenhouse conditions. Table S6. Two-way analysis of variance (ANOVA) of the effects of Se, P treatment as well as their interactions on the dry matter weight of winter wheat (Triticum aestivum cv Bainong 207) grown under greenhouse conditions. Table S7. Two-way analysis of variance (ANOVA) of the effects of Se, P treatment as well as their interactions on the root morphology of winter wheat (Triticum aestivum cv Bainong 207) grown under greenhouse conditions. Table S8. Two-way analysis of variance (ANOVA) of the effects of Se, P treatment as well as their interactions on the photosynthetic parameter of winter wheat (Triticum aestivum cv Bainong 207) grown under greenhouse conditions. [file 12870_2023_4227_MOESM1_ESM.docx]

**Supporting Information Tables S1–S8**

**Table S1** Two-way analysis of variance (ANOVA) of the effects of Se, P treatment as well as their interactions on the P concentration and accumulation of winter wheat (*Triticum aestivum* cv Bainong 207) grown under greenhouse conditions.

| Source of Variation (Treatment) |  | DF | |  | Shoot P concentration | | |  | Root P concentration | | |
| --- | --- | --- | --- | --- | --- | --- | --- | --- | --- | --- | --- |
|  |  |  |  |  | F |  | P |  | F |  | P |
| Se treatment |  | 2 | |  | 16.5 |  | 0.000 |  | 1.17 |  | 0.334 |
| P treatment |  | 2 | |  | 599 |  | 0.000 |  | 42.6 |  | 0.000 |
| Se×P |  | 4 | |  | 15.6 |  | 0.000 |  | 9.13 |  | 0.000 |
| Source of Variation (Treatment) |  | | DF |  | Shoot P accumulation | | |  | Root P accumulation | | |
|  |  |  |  |  | F |  | P |  | F |  | P |
| Se treatment  P treatment  Se×P |  | 2 | |  | 42.0 |  | 0.000 | 2.41 | |  | 0.118 |
|  |  | 2 | |  | 661 |  | 0.000 |  | 18.5 |  | 0.000 |
|  |  | 4 | |  | 20.8 |  | 0.000 |  | 20.0 |  | 0.000 |

**Table S2** Two-way analysis of variance (ANOVA) of the effects of Se, P treatment as well as their interactions on the Se concentration and accumulation of winter wheat (*Triticum aestivum* cv Bainong 207) grown under greenhouse conditions.

| Source of Variation (Treatment) |  | | DF |  | Shoot Se concentration | | |  | Root Se concentration | | |  |
| --- | --- | --- | --- | --- | --- | --- | --- | --- | --- | --- | --- | --- |
|  |  | |  |  | F |  | P |  | F |  | P |  |
| Se treatment |  | | 2 |  | 86717 |  | 0.000 |  | 159904 |  | 0.000 |  |
| P treatment |  | | 2 |  | 927 |  | 0.000 |  | 7048 |  | 0.000 |  |
| Se×P |  | | 4 |  | 555 |  | 0.000 |  | 3470 |  | 0.000 |  |
| Source of Variation (Treatment) |  | DF | |  | Shoot Se accumulation | | |  | Root Se accumulation | | |  |
|  |  |  |  |  | F |  | P |  | F |  | P |  |
| Se treatment  P treatment  Se×P |  | | 2 |  | 4241 |  | 0.000 | 8592 | |  | 0.000 |  |
|  |  | | 2 |  | 26.5 |  | 0.000 |  | 636 |  | 0.000 |  |
|  |  | | 4 |  | 72.8 |  | 0.000 |  | 179 |  | 0.000 |  |

**Table S3** Two-way analysis of variance (ANOVA) of the effects of Se, P treatment as well as their interactions on the P and Se migration coefficient of winter wheat (*Triticum aestivum* cv Bainong 207) grown under greenhouse conditions.

| Source of Variation (Treatment) |  | DF |  | P migration coefficient | | |  | Se migration coefficient | | |  |
| --- | --- | --- | --- | --- | --- | --- | --- | --- | --- | --- | --- |
|  |  |  |  | F |  | P |  | F |  | P |  |
| Se treatment |  | 2 |  | 23.6 |  | 0.000 |  | 473 |  | 0.000 |  |
| P treatment |  | 2 |  | 3.06 |  | 0.072 |  | 27.6 |  | 0.000 |  |
| Se×P |  | 4 |  | 6.80 |  | 0.002 |  | 27.5 |  | 0.000 |  |

**Table S4** Two-way analysis of variance (ANOVA) of the effects of Se, P treatment as well as their interactions on the subcellular fractions of Se in tissues of winter wheat (*Triticum aestivum* cv Bainong 207) grown under greenhouse conditions.

| Source of Variation (Treatment) |  | DF |  | Shoot | | | | | | | | | | | | |
| --- | --- | --- | --- | --- | --- | --- | --- | --- | --- | --- | --- | --- | --- | --- | --- | --- |
|  |  |  |  | Cell wall | | | Cell organelle | | | | | Soluble fraction | | | | |
|  |  |  |  | F |  | P | |  | F |  | P | |  | F |  | P |
| Se treatment |  | 2 |  | 1959 |  | 0.000 | |  | 3619 |  | 0.000 | |  | 648 |  | 0.000 |
| P treatment |  | 2 |  | 23.5 |  | 0.000 | |  | 26.5 |  | 0.000 | |  | 53.4 |  | 0.000 |
| Se×P |  | 4 |  | 14.5 |  | 0.000 | |  | 11.3 |  | 0.000 | |  | 34.9 |  | 0.000 |
| Source of Variation (Treatment) |  | DF |  | Root | | | | | | | | | | | | |
|  |  |  |  | Cell wall | | | Cell organelle | | | | | Soluble fraction | | | | |
|  |  |  |  | F |  | P | |  | F |  | P | |  | F |  | P |
| Se treatment |  | 2 |  | 1568 |  | 0.000 | |  | 359 |  | 0.000 | |  | 409 |  | 0.000 |
| P treatment |  | 2 |  | 242 |  | 0.000 | |  | 299 |  | 0.000 | |  | 220 |  | 0.000 |
| Se×P |  | 4 |  | 73.7 |  | 0.000 | |  | 272 |  | 0.000 | |  | 169 |  | 0.000 |

**Table S5** Two-way analysis of variance (ANOVA) of the effects of Se, P treatment as well as their interactions on Se species in tissues of winter wheat (*Triticum aestivum* cv Bainong 207) grown under greenhouse conditions.

| Source of Variation (Treatment) |  | DF |  | Shoot | | | | | | | | | | | |
| --- | --- | --- | --- | --- | --- | --- | --- | --- | --- | --- | --- | --- | --- | --- | --- |
|  |  |  |  | Se (IV) | | | Se (VI) | | | | | SeCys_2_ | | | |
|  |  |  |  | F |  | P | |  | F |  | P |  | F |  | P |
| Se treatment |  | 2 |  | 764 |  | 0.000 | |  | 545 |  | 0.000 |  | 126 |  | 0.000 |
| P treatment |  | 2 |  | 3.07 |  | 0.071 | |  | 24.6 |  | 0.000 |  | 0.986 |  | 0.392 |
| Se×P |  | 4 |  | 3.07 |  | 0.043 | |  | 24.6 |  | 0.000 |  | 2.71 |  | 0.063 |
| Source of Variation (Treatment) |  | DF |  | Shoot | | | | | | | | | | | |
|  |  |  |  | MeSeCys | | | SeMet | | | | |  | | | |
|  |  |  |  | F |  | P | |  | F |  | P |  | | | |
| Se treatment |  | 2 |  | 840 |  | 0.000 | |  | 8146 |  | 0.000 |  |  |  |  |
| P treatment |  | 2 |  | 6.63 |  | 0.007 | |  | 1259 |  | 0.000 |  |  |  |  |
| Se×P |  | 4 |  | 5.31 |  | 0.005 | |  | 441 |  | 0.000 |  |  |  |  |
| Source of Variation (Treatment) |  | DF |  | Root | | | | | | | | | | | |
|  |  |  |  | Se (IV) | | | Se (VI) | | | | | SeCys_2_ | | | |
|  |  |  |  | F |  | P | |  | F |  | P |  | F |  | P |
| Se treatment |  | 2 |  | 104 |  | 0.000 | |  | 124 |  | 0.000 |  | 415 |  | 0.000 |
| P treatment |  | 2 |  | 0.087 |  | 0.434 | |  | 15.3 |  | 0.000 |  | 24.9 |  | 0.000 |
| Se×P |  | 4 |  | 5.94 |  | 0.003 | |  | 15.3 |  | 0.000 |  | 325 |  | 0.000 |
| Source of Variation (Treatment) |  | DF |  | Root | | | | | | | | | | | |
|  |  |  |  | MeSeCys | | | SeMet | | | | |  | | | |
|  |  |  |  | F |  | P | |  | F |  | P |  |  |  |  |
| Se treatment |  | 2 |  | 2189 |  | 0.000 | |  | 1235 |  | 0.000 |  |  |  |  |
| P treatment |  | 2 |  | 69.5 |  | 0.000 | |  | 75.5 |  | 0.000 |  |  |  |  |
| Se×P |  | 4 |  | 47.6 |  | 0.000 | |  | 25.0 |  | 0.000 |  |  |  |  |

**Table S6** Two-way analysis of variance (ANOVA) of the effects of Se, P treatment as well as their interactions on the dry matter weight of winter wheat (*Triticum aestivum* cv Bainong 207) grown under greenhouse conditions.

| Source of Variation (Treatment) |  | DF |  | Dry matter weight of shoot | | |  | Dry matter weight of root | | |
| --- | --- | --- | --- | --- | --- | --- | --- | --- | --- | --- |
|  |  |  |  | F |  | P |  | F |  | P |
| Se treatment |  | 2 |  | 43.6 |  | 0.000 |  | 7.17 |  | 0.005 |
| P treatment |  | 2 |  | 187 |  | 0.000 |  | 97.3 |  | 0.000 |
| Se×P |  | 4 |  | 53.0 |  | 0.000 |  | 60.0 |  | 0.000 |

**Table S7** Two-way analysis of variance (ANOVA) of the effects of Se, P treatment as well as their interactions on the root morphology of winter wheat (*Triticum aestivum* cv Bainong 207) grown under greenhouse conditions.

| Source of Variation (Treatment) |  | DF | Root Length | | | |  | | \| Root Total Surface Area \| \| --- \| | | | | |  |
| --- | --- | --- | --- | --- | --- | --- | --- | --- | --- | --- | --- | --- | --- | --- | --- |
|  |  |  |  | F |  | P |  | F | | |  | P | | |
| Se treatment |  | 2 |  | 166 |  | 0.000 |  | 4.92 | | |  | 0.020 | | |
| P treatment |  | 2 |  | 1684 |  | 0.000 |  | 18.3 | | |  | 0.000 | | |
| Se×P |  | 4 |  | 338 |  | 0.000 |  | 5.28 | | |  | 0.005 | | |
| Source of Variation (Treatment) |  | DF |  | Root Volume | | |  | Average Root Diameter | | | | | | |
|  |  |  |  | F |  | P |  | F | | |  | P | | |
| Se treatment |  | 2 |  | 52.4 |  | 0.000 |  | 31.1 | | |  | 0.000 | | |
| P treatment |  | 2 |  | 83.8 |  | 0.000 |  | 1.87 | | |  | 0.183 | | |
| Se×P |  | 4 |  | 36.3 |  | 0.000 |  | 35.5 | | |  | 0.000 | | |
| Source of Variation (Treatment) |  | DF |  | Root Tip number | | |  | Root Forks | | | | | | |
|  |  |  |  | F |  | P |  | F | |  | | | P | |
| Se treatment |  | 2 |  | 9260 |  | 0.000 |  | 6198 | | |  | | 0.000 | |
| P treatment |  | 2 |  | 2689 |  | 0.000 |  | 19411 | | |  | | 0.000 | |
| Se×P |  | 4 |  | 6534 |  | 0.000 |  | 12042 | | |  | | 0.000 | |

**Table S8** Two-way analysis of variance (ANOVA) of the effects of Se, P treatment as well as their interactions on the photosynthetic parameter of winter wheat (*Triticum aestivum* cv Bainong 207) grown under greenhouse conditions.

| Source of Variation (Treatment) |  | DF |  | Net Photosynthetic Rate | | |  | \| Stomatal Conductance \| \| --- \| | | |
| --- | --- | --- | --- | --- | --- | --- | --- | --- | --- | --- | --- |
|  |  |  |  | F |  | P |  | F |  | P |
| Se treatment |  | 2 |  | 9.61 |  | 0.001 |  | 178 |  | 0.000 |
| P treatment |  | 2 |  | 134 |  | 0.000 |  | 501 |  | 0.000 |
| Se×P |  | 4 |  | 10.9 |  | 0.000 |  | 72.4 |  | 0.000 |
| Source of Variation (Treatment) |  | DF |  | Intercellular CO_2_ | | |  | Transpiration Rate | | |
|  |  |  |  | F |  | P |  | F |  | P |
| Se treatment |  | 2 |  | 495 |  | 0.000 |  | 65.7 |  | 0.000 |
| P treatment |  | 2 |  | 468 |  | 0.000 |  | 239 |  | 0.000 |
| Se×P |  | 4 |  | 241 |  | 0.000 |  | 36.1 |  | 0.000 |
